# The road to commercialization in Africa: lessons from developing the sickle-cell drug Niprisan

**DOI:** 10.1186/1472-698X-10-S1-S11

**Published:** 2010-12-13

**Authors:** Kumar Perampaladas, Hassan Masum, Andrew Kapoor, Ronak Shah, Abdallah S Daar, Peter A Singer

**Affiliations:** 1 McLaughlin-Rotman Centre for Global Health, University Health Network and University of Toronto, 101 College Street Suite 406, Toronto ON, M5G 1L7, Canada

## Abstract

**Background:**

Developing novel drugs from traditional medicinal knowledge can serve as a means to improve public health. Yet countries in sub-Saharan Africa face barriers in translating traditional medicinal knowledge into commercially viable health products. Barriers in moving along the road towards making a new drug available include insufficient manufacturing capacity; knowledge sharing between scientists and medical healers; regulatory hurdles; quality control issues; pricing and distribution; and lack of financing. The case study method was used to illustrate efforts to overcome these barriers during the development in Nigeria of Niprisan – a novel drug for the treatment of sickle cell anemia, a chronic blood disorder with few effective therapies.

**Discussion:**

Building on the knowledge of a traditional medicine practitioner, Nigeria’s National Institute for Pharmaceutical Research and Development (NIPRD) developed the traditional herbal medicine Niprisan. The commercialization of Niprisan reached a number of commercial milestones, including regulatory approval in Nigeria; securing US-based commercial partner XeChem; demonstrating clinical efficacy and safety; being awarded orphan drug status by the US Food and Drug Administration; and striking important relationships with domestic and international groups. Despite these successes, however, XeChem did not achieve mainstream success for Niprisan in Nigeria or in the United States. A number of reasons, including inconsistent funding and manufacturing and management challenges, have been put forth to explain Niprisan’s commercial demise. As of this writing, NIPRD is considering options for another commercial partner to take the drug forward.

**Summary:**

Evidence from the Niprisan experience suggests that establishing benefit-sharing agreements, fostering partnerships with established research institutions, improving standardization and quality control, ensuring financial and managerial due diligence, and recruiting entrepreneurial leaders capable of holding dual scientific and business responsibilities should be incorporated into future drug development initiatives based on traditional medicines. Country-level supporting policies and conditions are also important. With more experience and support, and an improved environment for innovation, developing new drugs from traditional medicines may be an attractive approach to addressing diseases in sub-Saharan Africa and other regions.

## Background

Bringing medicines to market for diseases that affect public health can be a challenging task, especially in developing countries where the local environment for innovation contains challenges that include technical capacity, financing, and entrepreneurship [1]. But promoting health innovation in developing countries offers opportunities for these nations to tackle endemic public health issues, and such endeavors have the potential to create a reliable stream of affordable medicines for local diseases [2,3]. (See the introductory paper in this BMC series for further discussion of the innovation context.)

In countries such as China and India, traditional herbal medicines have an important role in public health, as these medicines are frequently used as a first line of therapy for meeting the health needs of the population [4-8]. This role is attributed to the widespread acceptance of traditional medicine, its strong link to cultural beliefs, affordability, and confidence in traditional medicine practitioners [8-13]. Given the innovative potential of traditional medicines and opportunities for first-in-class therapies, the governments of countries like China have established innovation policies that aim to build capacity in their commercialization [7].

Similar to Asian countries, the prevalent use of indigenous traditional medicines in Africa has prompted a number of sub-Saharan African nations to establish research institutes aimed at the study of traditional medicines [12-15]. (For further discussion on country-specific use of traditional medicines, see the country papers in this BMC series.) Yet, pursuing commercial development to capitalize on indigenous knowledge of traditional medicines in sub-Saharan Africa faces a number of challenges, including difficulty in building knowledge-sharing partnerships between conventional scientists and traditional medical healers; overcoming problems with quality control; accurately characterizing raw materials; financing; and a lack of appetite for high-risk biotechnology ventures [1-3,16]. Addressing these barriers is pertinent for supporting commercialization, as is identifying policy interventions that could ensure the mobilization of sub-Saharan Africa’s pharmaceutical potential. This paper illustrates efforts to overcome these barriers in the development of Niprisan in Nigeria – a novel drug for the treatment of sickle cell anemia [17,18].

We used a case study design. We conducted interviews with informed consent of key personnel of NIPRD and Xechem International (Xechem) between 2007 and 2009, including senior directors at NIPRD and the then-CEO of Xechem. Literature analysis sources included background documents on Niprisan, related articles from peer-reviewed literature, news reports, books, financial filings, web sites, and relevant reports from the World Health Organization and other groups. Representatives of NIPRD and Xechem were asked to fact-check the case study; the analysis and interpretation is our own. All quotes are from the interviews unless noted, and with permission. This study was approved by the Office of Research Ethics of the University of Toronto.

In this article, we chronicle the development of Niprisan. We begin by describing how Niprisan was discovered, proceed to discuss the partnerships and commercial strategy undertaken, and note achievements and shortcomings with a view to distilling lessons for the commercialization of medicines in sub-Saharan Africa.

## Discussion

### The discovery of Niprisan

Sickle cell anemia, a genetic condition that primarily affects those of African ancestry, significantly reduces the patient’s quality of life and productivity; the condition causes episodes of pain that can last for hours or days at time [17-20]. The disease interferes with the body’s oxygen transfer process, and can lead to the formation of small blood clots, which over time deprive organs and tissues of oxygen. Nigeria is home to the largest population of sickle cell anemia patients, estimated to be in excess of 4 million patients with more than 150 000 children born annually with the disease [19]. The condition is also incident in the African diaspora population in North America and Europe.

Despite decades of research, only one FDA approved drug, hydroxyurea, is available for use in sickle cell anemia [18]. This chemotherapeutic agent stimulates healthy production of fetal hemoglobin to counteract the sickling process [18]. Although long-term clinical studies have shown the drug to improve survival, concerns over genotoxicity and carcinogenesis remain [17,18].

In the late 1980s, in an effort to advance research of traditional herbal medicines, Nigeria’s Ministry of Science and Technology established the NIPRD – a research body dedicated to identifying, characterizing, developing, and documenting the use of traditional herbal medicines in Nigeria. Despite the frequent use of traditional medical healers by Nigeria’s populace, tapping into their knowledge remained a challenge, due to a prevalent lack of trust between conventional scientists and traditional medical healers and concerns over bio-piracy [11,13].

In order to overcome this barrier and build an adequate pipeline of traditional herbal medicines, NIPRD implemented benefit-sharing agreements with traditional herbal healers – a measure that was seen by Dr. Charles Wambebe, the first director of NIPRD, to be necessary for brokering trust and gaining access to a vast pool of traditional medical knowledge [21]. The agreements formally involved the traditional practitioner in the development program, and entitled them to royalties on the sale of any commercial product developed with their proprietary knowledge [21]. In exchange, NIPRD was to receive all necessary information pertaining to the traditional herbal medicine, including its medicinal use, method of collection, and method of manufacture [21].

Niprisan was brought to the attention of NIPRD by a local reverend, Paul Ogunyale, in 1992. The Reverend, who also holds master’s degree, claimed to be using Niprisan to treat members of his congregation for sickle cell anemia. NIPRD signed a formal benefit-sharing agreement with Reverend Ogunyale, which entitled him and his family to future royalties on Niprisan in exchange for the recipe of Niprisan and disclosure of all trade secrets [21]. Preliminary testing and experimentation by NIPRD researchers and Paul Ogunyale identified several raw materials thought to be responsible for the beneficial effect; these sources included *Piper guineenses* seeds*, Pterocapsus osum* stems*, Eugenia caryophyllum* fruit*, and Sorghum bicolor* leaves [22,23].

According to Dr. Wambebe, the agreement with Reverend Ogunyale and NIPRD was considered to be the first benefit sharing arrangement of its kind in Nigeria.

### Partnering with the Children’s Hospital of Philadelphia

In 1997, the scientists at NIPRD sent samples of Niprisan to Dr. Toshio Asakura of the Children’s Hospital of Philadelphia. Dr. Asakura, an expert in sickle cell anemia disorders, conducted animal studies to test the efficacy of Niprisan [24-27]. The results of his work demonstrated both in transgenic mice and in patient samples with sickle cell disease that Niprisan did have potential anti-sickling properties [24-27].

In addition to the findings by Dr. Asakura’s group, NIPRD conducted clinical studies to determine Niprisan’s efficacy in patients with sickle cell anemia. Findings from these studies conducted at hospitals in Nigeria showed that the drug significantly reduced the number of vaso-occlusive crises [25-27]. The clinical studies conducted by NIPRD also reported that Niprisan had an extremely low toxicity profile, unlike other drugs that have been developed for sickle cell anemia [25-27].

NIPRD’s research efforts resulted in a United States patent being filed in 1998 entitled: *Piper guineense, Pterocarpus osun, Eugenia caryophyllata, and Sorghum bicolor extracts for treating sickle cell disease *[23].

### Partnering with Xechem International (Xechem)

According to scientists at NIPRD, finding a commercial partner in Nigeria to scale up and manufacture Niprisan proved difficult as the local pharmaceutical industry in Nigeria has traditionally focused on marketing and distribution, with limited capacity in formulation and development. According to Dr. Inyang, currently the Director General of NIPRD: “The local drug industry is not geared towards natural product manufacturing. They only produce imported generics. At the time, nobody had the facilities to produce Niprisan. As a result, I guess they [private sector] saw it as a very expensive investment with a lot of uncertainty...”

After approaching several pharmaceutical firms in Nigeria including Emzor Pharmaceuticals – one of Nigeria’s leading pharmaceutical manufacturers – the challenge of finding a partner with commercial interest and facilities remained. But in the fall of 2001, as part of Nigeria’s effort to increase research collaborations in biotechnology with research institutes abroad, Dr. Turner T. Isoun, a senior member of Nigeria’s Federal Ministry of Science and Technology, led a delegation to Rutgers University for a conference to explore opportunities for research collaborations [28]. It was at this meeting that members of the Nigerian delegation met Dr. Ramesh Pandey, CEO of US-based Xechem International and an experienced bio-entrepreneur with expertise in pharmaceutical manufacturing. Dr. Pandey’s affiliation with Rutgers University dated back to the 1980s when Dr. Pandey was a Visiting Professor at the Waksman Institute of Microbiology; at the time of the delegation’s visit, Dr. Pandey’s firm was collaborating with the university on various research projects [28]. Dr. Isoun offered Dr. Pandey an invitation to Nigeria to explore possible collaboration in biotechnology.

After visiting Nigeria and meeting with officials at NIPRD, Dr Pandey became aware of Niprisan’s potential to treat sickle-cell anemia, and decided to take the opportunity to lead Niprisan’s commercialization program. On July 18^th^ 2002, Xechem was granted an exclusive license to commercialize Niprisan for the treatment of sickle cell anemia. In exchange, NIPRD reportedly received a 7.5% royalty rate of gross sales, and an up-front cash payment of $115,000 [21].

### Commercial strategy

According to our interviews with Dr. Pandey and members of the NIPRD, Niprisan was estimated to have a market potential of tens of millions of dollars annually in Nigeria alone, and Xechem reportedly intended to sell a month’s supply of the drug for $20-$25 USD.

The decision to produce Niprisan locally was a stipulation laid out in the licensing agreement between Xechem and NIPRD. Given the sales potential, Nigeria’s comparative advantage in raw materials, and land for the manufacturing facility provided free of cost by the Nigerian government, the advantages of being located in Nigeria outweighed concerns over manufacturing capabilities, according to Dr. Pandey and other sources [21].

Our interviews with Dr. Pandey indicated that Xechem intended to make Niprisan available in the United States by leveraging the orphan drug designation program offered by the US Food and Drug Administration. This designation was sought by Dr. Pandey, who submitted an application after learning that sickle cell anemia is listed as an orphan drug disease in the United States – one from which a percentage of African-Americans suffer, who would be potential users of this treatment. Orphan drug status was granted in 2003 and brought Xechem advantages including waiving of regulatory fees, additional funding, and an increase in investor confidence [29-31].

### Launch – and crash

In July of 2006, the National Agency for Food and Drug Administration and Control approved Niprisan for sale in Nigeria. In a strong display of government support, the Nigerian President Olusegun Obasanjo was present at the official launch of *Nicosan* (the brand name of Niprisan) in Nigeria [28].

However, revenues generated from small-scale production were disappointing: the drug generated revenue totaling $197,000 as of December 31, 2006 [30]. According to our interviews, sufficient demand for the product was evident, but production bottlenecks including raw material supply and scaling up manufacturing began to arise. Further, according to Xechem International’s 2006 annual report, its operating losses as of December 31, 2005 and 2006 were in excess of $4 million [30]. Concerns rose over the financial and operational stability of Xechem, despite Xechem securing nearly $9 million in loan commitments from US and Nigerian banks to fund the construction of the manufacturing facility in Nigeria [32-35].

In December 2006, Xechem International announced the appointment of Mr. Iretiolu Oniyide, a Nigerian with a banking background, to handle the day-to-day operations of Xechem in Nigeria and to carry forward the commercialization of Nicosan in Nigeria [30-35]. In July of 2007, Xechem announced a plan to reduce costs, which included closing down their US operations and increasing its operational capacity in Nigeria [30-35]. In addition to new cost cutting measures, Dr. Robert Swift became the chief executive officer of Xechem International, replacing Dr. Pandey [30-35]. Despite the financial and structural changes, Xechem International filed for bankruptcy in the United States in late 2008, and in early 2009 the Nigerian government revoked the license for Niprisan [34].

### Lessons learned

Niprisan’s commercial demise has been attributed to multiple factors, including a lack of cash flow, management missteps, challenges in manufacturing and product development, and a timeline to large-scale manufacturing that was longer and more difficult than anticipated. We examine five key lessons that can be drawn from the case of Niprisan to illustrate how policy-makers might support entrepreneurs and domestic innovation in Nigeria, and more broadly in sub-Saharan Africa and the developing world.

#### Support business-friendly environments

According to the World Bank, Nigeria ranks poorly when compared to OECD countries in parameters that include dealing with construction permits, enforcing contracts, starting a business, registering property, and trading across borders [36]. Some of these issues were evident in the development of Niprisan, as Xechem experienced delays in securing construction permits and importing equipment. As Dr. Pandey recalls, “one of the biggest hurdles in operating Xechem Nigeria was the difficulty in getting day-to-day activities done. It is very difficult in Nigeria.” To improve the local business environment, especially when firms are working with international partners, governments should aim to reduce the time firms spend getting approval for services that have a direct impact on business performance, i.e. importing/exporting, customs tariffs, and land permits.

#### Broker benefit-sharing agreements with traditional medical healers

To increase knowledge sharing between traditional medical healers and conventional scientists, and potentially tap into a new source of drug products, NIPRD implemented benefit-sharing agreements that allowed a traditional medical healer to participate in the profits of any drug originating from their recipe. Such agreements allowed NIPRD to foster relationships with traditional medical healers across Nigeria, in an effort to identify promising treatments for further development. According to Dr. Wambebe, one of the aims of the benefit-sharing agreements was to engage the stakeholders as much as possible in the process, ensuring that everyone was both motivated and vested in the success of the product. In the case of Niprisan, Reverend Ogunyale was involved in the development work of Niprisan via a staff position at NIPRD, which also led to a publication [27].

Prior to the establishment of NIPRD, many traditional remedies used by local healers went undocumented. In the case of Niprisan, NIPRD relied on Reverend Ogunyale for his knowledge of the plants and herbs used in the formulation of Niprisan. To be successful in supporting domestic pharmaceutical drug development, policy-makers should consider benefit-sharing agreements as a model for sourcing new medicines, by bringing together conventional scientists with traditional medical healers in a fair and equitable manner. (For another institution that has successfully implemented benefit-sharing in Africa, see the paper on IMRA in this BMC series.)

#### Expand incentives for local innovation

Nigeria, like many developing countries, has limited capacity to provide broad health care coverage, including reimbursement of pharmaceuticals. While NIPRD’s partner Xechem forecasted millions of dollars in revenues, it may not have fully considered the implications of the fact that the majority of purchases would be out-of-pocket expenses for the consumer.

According to the directors at NIPRD, “many of the patients who were on Niprisan could no longer afford to continue taking the medication.” Prior to commercial production of Niprisan by Xechem, the price of the drug in Nigeria was reportedly set at $4 USD a month. After licensing Niprisan to Xechem, the price reportedly increased to $20-25 USD per month, which included dispensing fees from pharmacies. To ensure prices are kept affordable and incentives for local innovation remain, governments should continue to seek policies that address broad health coverage in Nigeria, in addition to expanding programs aimed to subsidize the cost of research and development. Without proper incentives to address price and cost of development, good alternative treatments developed from indigenous sources may be out of reach for the majority of the population.

#### Improve standardization and quality control

Despite being approved by the National Agency for Food and Drug Administration and Control in Nigeria, Xechem experienced quality control issues. According to Xechem’s 2006 annual report, securing and sourcing the correct raw materials for the preparation proved to be challenging: “there is a lack of data to document the influence of raw materials (i.e. plant material quality, age, time of harvest, location, soil quality, preparation, handling, etc…) on the production of NICOSAN” (Niprisan) [31]. Xechem acknowledged the difficulty in establishing a consistent formulation of the drug, an issue that was raised by the US Food and Drug Administration and Dr. Asakura.

Establishing quality control is the most important barrier to success, says Dr. Inyang:

You should employ people that are trained and qualified in botanical research. They need to know exactly what they are doing, because there is a lot of variability in plants and herbs, and accurately sourcing the raw materials is critical for consistency…. you should be able to standardize the product, that’s the key word – standardize. Without that, it will undermine the value of traditional medicines, because any variation will affect the potency.

Despite Dr. Pandey’s expertise in pharmaceutical development, securing personnel to handle manufacturing proved to be difficult. One means to reduce production bottlenecks for firms looking to build manufacturing capacity in Nigeria might be to create applied training programs and internships in pharmaceutical manufacturing and quality control. The importance of training and on-the-job experience is further echoed by Dr. Inyang, who says “If you don’t have the money to buy the equipment, do the quality control, and train your workforce, then you are going to run into trouble.”

#### Foster partnerships to fill gaps in knowledge and technical expertise

Local production does provide advantages, but government agencies should also consider the challenges of the local environment and how it may affect collaborations. The partnering processes that led to the selection of Xechem as the commercial partner provides lessons for government agencies wishing to support local innovation, and for drug developers who need to manage the partnership. Government agencies must balance the potential economic gains of establishing a local pharmaceutical industry with the realities of the necessary skills and resources needed to support drug development in a resource-constrained environment.

NIPRD is a research institute focused on studying traditional herbal medicines. Recognizing the importance of having experienced collaborators with expertise in sickle cell anemia and drug development, NIPRD opted to collaborate with Children’s Hospital of Philadelphia and Dr. Pandey, respectively. Each of these collaborations contributed to reaching important milestones in the development of Niprisan, with the Children’s Hospital of Philadelphia conducting animal studies confirming the potential anti-sickling activity of Niprisan, and Dr. Pandey being primarily responsible for handling the manufacturing aspects of Niprisan.

For NIPRD, “a kind of multi-disciplinary collaboration is needed throughout the research and development process” says Dr. Wambebe. Multi-disciplinary collaborations, such as the partnerships with Rutgers University and Children’s Hospital of Philadelphia, will help scientists and entrepreneurs in countries such as Nigeria to address technological or learning gaps that would otherwise derail a drug development program.

Once a partnership is made, it must also be managed. Risks need to be understood and financial and managerial due diligence done by all partners. While every partnership is based on trust, clear milestones along with checks and balances may highlight problems early and increase the probability of success.

#### Engage skilled entrepreneurial leaders

According to our interviews with members of NIPRD, the lack of bio-entrepreneurs willing and able to enter high-risk capital-intensive biotechnology ventures is a problem in Nigeria. Despite this, the role of Drs. Wambebe, Inyang, and Pandey along with the team at NIPRD illuminates an important resource in cultivating Southern innovation: leadership.

In our interviews with Dr. Wambebe, he said “the most important thing I learned from this experience is the need for a champion to lead the process and make it happen – someone who can manage the partnerships, recruit talented professionals, approach government for funding, and handle the missteps and breakthroughs that go along with early stage drug development.” Dr. Wambebe lobbied the Nigerian government for funds needed to conduct the early development work and pilot clinical studies of Niprisan, and utilized funding sources such as the United Nations Development Program to upgrade the NIPRD facilities.

Similarly, Dr. Pandey played a critical role in achieving significant milestones for Niprisan. He raised financing commitments from US and Nigerian governments, negotiated a land deal, constructed a pilot manufacturing facility capable of producing smaller quantities of Niprisan, and spearheaded the granting of orphan drug status for Niprisan.

Bio-entrepreneurs in resource-constrained environments may need to balance technical and business responsibilities, and as Dr. Wambebe terms it act as the “internal champion.” Because of the challenging environment, firms need to find strong leadership that can hold dual scientific and business roles. Policy-makers should likewise be mindful of the importance of leadership to mitigate the risk of pitfalls; project management and stakeholder engagement are also important. Additional efforts to provide business and leadership training are warranted, whether through educational programs, on-the job training, or mentorship linkages with experienced professionals domestically or internationally.

## Summary

One of the key facts to emphasize about Niprisan is that it represents a novel drug for sickle-cell anemia, which is a serious problem in both Africa and the United States. Thus, the stakes are high. Although the first commercialization attempt of Niprisan did not succeed in making the drug widely available, the drug development process up to this stage embodied a number of significant achievements. As of this writing, NIPRD is considering options for another commercial partner to take the drug forward.

Despite these challenges, countries in sub-Saharan Africa have previously participated in the development of drugs from traditional medicines, illustrated by the isolation of vincristine from *Catharanthus roseus* (an indigenous plant to Madagascar used as an anti-cancer agent [37]) and the use of *Harpagophytum procumbens* (Devil’s Claw) to treat rheumatoid arthritis and inflammation [12]. There are plans for more systematic drug development support through initiatives such as the African Network for Drugs and Diagnostics Innovation (ANDI) [38].

To effectively capitalize on the knowledge of traditional medical healers and researchers alike, future innovation efforts need to incorporate broader actions aimed at providing incentives for the private sector and entrepreneurs, improving regulatory capacity, facilitating international partnerships for technology transfer, and brokering benefit sharing agreements between traditional medical healers and conventional scientists. Risks of the commercialization process also need to be understood by all partners, and financial and managerial due diligence ensured.

Building the capacity to produce novel and affordable medicines for local health problems may stimulate economic development, decrease dependency on international donor programs, and contribute to improving global health. Looking forward, sharing findings and lessons from cases such as Niprisan will help remove barriers on the road to commercialization in Africa. With more experience and support, future drug development efforts in Africa may increase their chances of success.

## Competing interests

None declared.

## Authors' contributions

Each author contributed to the concept and design of this study, participated in the site visit, analyzed the findings and participated in manuscript development.
